# Antigen Discovery in Circulating Extracellular Vesicles From *Plasmodium vivax* Patients

**DOI:** 10.3389/fcimb.2021.811390

**Published:** 2022-01-24

**Authors:** Iris Aparici-Herraiz, Melisa Gualdrón-López, Carlos J. Castro-Cavadía, Jaime Carmona-Fonseca, María Fernanda Yasnot, Carmen Fernandez-Becerra, Hernando A. del Portillo

**Affiliations:** ^1^ ISGlobal, Hospital Clínic - Universitat de Barcelona, Barcelona, Spain; ^2^ Grupo de Salud y Comunidad Cesar Uribe Piedrahíta, Universidad de Antioquia, Medellín, Colombia; ^3^ Grupo de Investigaciones Microbiológicas y Biomédicas de Córdoba-GIMBIC, Universidad de Córdoba, Monteria, Colombia; ^4^ Institut d’Investigació en Ciències de la Salut Germans Trias i Pujol, Badalona, Spain; ^5^ CIBER de Enfermedades Infecciosas (CIBERINFEC), Barcelona, Spain; ^6^ Institució Catalana de Recerca i Estudis Avançats (ICREA), Barcelona, Spain

**Keywords:** *Plasmodium vivax*, extracellular vesicles, antigen discovery, direct immuno-affinity capture, proteomics

## Abstract

*Plasmodium vivax* is the most widely distributed human malaria parasite with 7 million annual clinical cases and 2.5 billion people living under risk of infection. There is an urgent need to discover new antigens for vaccination as only two vaccine candidates are currently in clinical trials. Extracellular vesicles (EVs) are small membrane-bound vesicles involved in intercellular communication and initially described in reticulocytes, the host cell of *P. vivax*, as a selective disposal mechanism of the transferrin receptor (CD71) in the maturation of reticulocytes to erythrocytes. We have recently reported the proteomics identification of *P. vivax* proteins associated to circulating EVs in *P. vivax* patients using size exclusion chromatography followed by mass spectrometry (MS). Parasite proteins were detected in only two out of ten patients. To increase the MS signal, we have implemented the direct immuno-affinity capture (DIC) technique to enrich in EVs derived from CD71-expressing cells. Remarkably, we identified parasite proteins in all patients totaling 48 proteins and including several previously identified *P. vivax* vaccine candidate antigens (MSP1, MSP3, MSP7, MSP9, Serine-repeat antigen 1, and HSP70) as well as membrane, cytosolic and exported proteins. Notably, a member of the *Plasmodium* helical interspersed sub-telomeric (PHIST-c) family and a member of the *Plasmodium* exported proteins, were detected in five out of six analyzed patients. Humoral immune response analysis using sera from vivax patients confirmed the antigenicity of the PHIST-c protein. Collectively, we showed that enrichment of EVs by CD71-DIC from plasma of patients, allows a robust identification of *P. vivax* immunogenic proteins. This study represents a significant advance in identifying new antigens for vaccination against this human malaria parasite.

## Introduction

Of the five species causing human malaria, *Plasmodium falciparum*, *P. vivax*, *P. ovale*, *P. malariae* and *P. knowlesi*, *P. vivax* is the most widely distributed representing 53% of malaria burden in the South-East Asia region and the most predominant species in the region of the Americas (WHO, 2020). Recent estimates of its burden indicate that close to 3.3 billion of people are under risk of infection and near 7 million yearly clinical cases. Major biological differences between *P. vivax* and *P. falciparum*, the most virulent species, indicate that current available tools against *P. falciparum* will not work against *P. vivax*, thus challenging its elimination (Mueller et al., 2009; Howes et al., 2016). In fact, where effective control measurements are reducing the burden of *P. falciparum*, in these same areas, detection of *P. vivax* is rising-up (Price et al., 2020), and this includes sub-Saharan Africa where *P. vivax* was considered to be mostly absent (Twohig et al., 2019).

Among control tools, vaccines remain the most cost-effective public health measurement. Remarkably, WHO has recently announced the approval of the RTS,S (Mosquirix) as a vaccine against *P. falciparum* recommended for young children in Africa under moderate to high risk of transmission. In spite of this major historical achievement, this vaccine does not cross-protect against *P. vivax*, a species for which vaccine development lags well behind that of *P. falciparum*. In fact, only six vaccine candidates representing three antigens, the circumsporozoite surface protein (CSP), the Duffy binding protein (DFP) and the Ookinete surface protein (Pvs25) have progressed into human clinical trials (Draper et al., 2018; De et al., 2021). These data, strongly reinforce the need for discovering new antigens for vaccination and novel vaccine approaches against this neglected human malaria parasite.

Extracellular vesicles (EVs) are a heterogeneous group of double membrane particles that are secreted from live cells of all the three life kingdoms and have recently emerged as relevant mediators of intercellular communication (Yanez-Mo et al., 2015). EVs have been classified into two main categories, microvesicles and exosomes, based on their size, biogenesis and composition. Exosomes are endocytic vesicles that are released from most cell types by inward budding of multivesicular bodies and their size range from 40–100 nm. Microvesicles are generated by the budding of the plasma membrane and their size range from 100 to 1,000 nm (Thery, 2011; Raposo and Stoorvogel, 2013). Exosomes and microvesicles express markers of their parent cells, and are specifically enriched in molecules associated with their biogenesis pathway and those selectively packaged into them (Hanson and Cashikar, 2012).

Investigations into the various vesicle types, from both host and parasite origin, have revealed important roles for EVs in disease pathogenesis and susceptibility, as well as in cell–cell communication and immune responses (Marcilla et al., 2014; Robbins and Morelli, 2014; Sampaio et al., 2017). In malaria, the transfer of membrane and cytosolic proteins, lipids, DNA, and RNA through EVs modulate the immune response (Couper et al., 2010) and cellular communication between parasites including factors that triggers differentiation to transmission stages (Mantel et al., 2013; Regev-Rudzki et al., 2013). Importantly, for what we believe is the first time, the physiological role of EVs in malaria demonstrated that they facilitate intrasplenic infections in *P. vivax* (Toda et al., 2020), now known to represent the largest cryptic parasite biomass during chronic infections (Kho et al., 2021a; Kho et al., 2021b).

*P. vivax* preferentially, if not exclusively, invades reticulocytes, young red cells where exosomes were first described as a cargo-disposal mechanism that selectively remove proteins such as the transferrin receptor CD71 during the maturation of reticulocytes to erythrocytes (Harding et al., 1983; Pan and Johnstone, 1983). Using a reticulocyte-prone rodent *P. yoelli* malaria model, we previously demonstrated the presence of parasite proteins in plasma-derived EVs and showed that reticulocyte-derived exosomes from infected mice protected immunized animals against lethal *P. yoelii* XL infections (Martin-Jaular et al., 2011). Moreover, such protection was spleen-dependent and involved CD8 T cell mediated immune response (Martin-Jaular et al., 2016). Interestingly, reticulocyte-derived exosomes contain HLA-I molecules suggesting a potential capacity for antigen presentation (Raposo and Stoorvogel, 2013; Diaz-Varela et al., 2018). More recently, we also demonstrated the presence of *P. vivax* proteins associated to plasma-derived EVs from vivax malaria patients isolated by size-exclusion chromatography (SEC) and using mass spectrometry (Toda et al., 2020). While three proteins were unequivocally identified (PHISTc, MSP3.1, GAPDH), we detected them in only two out of ten patients.

Here, we have implemented immune-capture of CD71^+^-EVs from plasma of *P. vivax* patients. This strategy greatly improved the enrichment of reticulocyte-specific EV markers and the coverage of EV-associated parasite proteins detected by mass spectrometry. Many of the parasite proteins identified are known to be immunogenic during natural infections and some of them represent vaccine candidates. Thus, this study is a step-forward to identify new antigens for vaccination against this human malaria parasite.

## Material and Methods

### Malaria Patients and Healthy Donors

Plasma samples from *P. vivax*-infected patients (PV) were collected at Tierralta, Colombia. The local ethical committee of Universidad de Córdoba reviewed and approved these studies (Acta #007 mayo 22 de 2017). Plasma from healthy donors (HD) was obtained at the Hospital Germans Trias i Pujol (Badalona, Barcelona, Spain) after expressed consent from the donors. Plasma from first-infected *P. vivax* patients from Barcelona was collected at the Hospital Clinic under the approval of the Hospital´s committee (identification number 4228).

### Blood Collection and Plasma Processing

Three mL of peripheral blood were collected by venipuncture in citrate pre-treated tubes. Samples were centrifuged at 400 x g for 10 min at room temperature (RT). Plasma was collected and centrifuged twice at 2000 x g for 10 min at 4°C. Supernatant was recovered, aliquoted and frozen at -80°C. Frozen plasmas from Tierralta were shipped on dry-ice to Institut Germans Trias i Pujol (IGTP, Badalona, Spain). Plasma collected from HDs and primo-infected *P. vivax* patients was similarly processed.

### EVs Purification by Direct Immuno-Affinity Capture (DIC)

One ml of plasma was thawed on ice and immediately diluted 1:10 in cold PBS and subjected to ultracentrifugation at 120,000 x g for four hours in a TH-641 Swinging Bucket Rotor (Sorvall™ WX^+^, ThermoFisher Ultracentrifuge) to pellet total EVs. The resulting pelleted samples (P120) were resuspended in 300 μl cold PBS and protein quantified by BCA Protein Assay Kit (Pierce, ThermoFisher Scientific). All the following steps were also performed at 4°C. Magnetic beads from Dynabeads™ Antibody Coupling Kit (ThermoFisher 14311D) were conjugated to recombinant rabbit monoclonal anti-CD71 antibody (Abcam: ab214039) following manufacturer’s instructions. P120 fractions were incubated with anti-CD71 coupled-dynabeads at 4°C with nutation and rotation for 16 hours. Nutation was obtained by placing the tubes in a container containing a circular sponge placed inside in diagonal position. After incubation, flow-through was recovered and then EVs-dynabeads complexes washed twice with ice-cold PBS. Immediately after the last wash, captured EVs (DIC fraction) were resuspended in cell lysis buffer (20Mm Tris-HCl pH 7.5, 150mM NaCl, 1mM Na_2_EDTA, 1mM EGTA, 1% Triton X-100), sonicated 1X for 10 seconds and incubated 30 minutes on ice. Finally, protein concentration was determined using a BCA Protein Assay Kit.

### Western Blot (WB)

Samples (P120 fractions, flow-through and EV-lysates) were mixed with NuPAGE LDS Sample Buffer (4X) (Invitrogen, NP0007) with 1mM DTT (10X) to denature proteins in reducing conditions. Samples were heated at 95°C for 5 min, beads removed from the sample using a magnet and the lysate was used for immunoblot analysis. For that, equivalent protein amount of P120 fraction (Input), flowthrough and DIC fractions were separated on a 8% SDS-PAGE, transferred on to Hybond-C nitrocellulose membrane (Amersham) and blocked in blocking buffer (1 X PBS, 0.1% Tween-20, 5% milk powder) overnight. After three washes, blots were incubated for 1 h with recombinant rabbit monoclonal anti-CD71 (1:1500, ab214039) in antibody buffer (1X PBS, 0.1% Tween-20, 1% milk powder). Subsequently, blots were washed and incubated for 1 h with the Li-Cor IRDye-labeled secondary antibodies IRDye 680RD goat anti-rabbit (1:20.000, 925-68021). Blots were scanned and analyzed with the Odyssey quantitative western blot near-infrared system (Li-Cor Biosciences, Lincoln, NE, USA) having the intensity of 700 channel set up at 5. Images were edited using the software Image J (NIH).

### Reticulocyte Culture and huRex Isolation

Human cord blood samples were obtained from the Barcelona Blood and Tissue Bank (www.bancsang.net/), following approval for the protocol and informed consent by the Clinical Research Ethics Committee of Vall d’Hebron University Hospital [PR(CS)236/2017]. Human reticulocyte preparations were performed as previously described (Llora-Batlle et al., 2020), with some modifications. Briefly, samples were centrifuged at 1000 x g for 15 min (acceleration = 6, deceleration = 2) and plasma was removed. Cell pellets were washed with incomplete Roswell Park Memorial Institute (RPMI) 1640 Medium at 500 x g for 10 min and resuspended at 50% hematocrit with incomplete RPMI. Five milliliters of resuspended blood were carefully loaded on 6 ml of 70% Percoll (GE Healthcare) and centrifuged at 1200 x g for 15 min at 20°C (acceleration = 4, deceleration = 0). Reticulocytes concentrated in the Percoll interface were collected and washed three times with incomplete RMPI (centrifugations at 400 x g for 5 min). To deplete leukocytes, reticulocyte-enriched pellets were resuspended to a final volume of 900 µl with incomplete RPMI and incubated with 100 µl of CD45 MicroBeads (Miltenyi Biotec, 130-045-801) for 18 min at 4°C, mixing every 3 min. The cells were washed with incomplete culture media and passed through LS Columns (Miltenyi Biotec, 130-042-401) placed on a magnetic stand. Eluents, containing reticulocyte-enriched fractions were washed twice with incomplete culture media, and reticulocyte enrichment quantified by BCB/Giemsa staining and viability checked by trypan blue staining. The fraction containing enriched reticulocyte was re-suspended in exosome-depleted medium (RPMI supplemented with 0.5% human AB serum), transferred to a culture flask at 3% hematocrit and incubated at 37°C, 5% CO_2_ for 36h. After 36-hour incubation, the culture was collected and centrifuged at 1300 x g for 20 min at RT (acceleration = 9, deceleration = 9). Human reticulocyte-derived exosomes (huRex) used as positive control of CD71^+^ EVs in western blot analysis were obtained as described (Diaz-Varela et al., 2018). Reticulocyte culture supernatants were used as starting material in positive control CD71^+^ EVs immune-capture experiments.

### Mass Spectrometry Analysis

#### Samples Preparation and Liquid Chromatography Tandem Mass Spectrometry (LC-MS/MS)

Samples were processed for peptide digestion using the commercial kit (PreOmics) according to the manufacturer’s protocol adapted for samples containing <20 μg of protein with slight modifications. Briefly, CD71^+^ EVs dynabeads complexes were resuspended in 20 μl of PreOmics lysis buffer and heated at 70°C for 10 minutes. After this step, samples were placed in a magnet and EV lysates recovered and loaded into the cartridge and treated with 20 μl of Digest buffer at 37°C, 500 r.p.m for 3h. Digested samples were washed and eluted according the standard protocol. After elution, samples were immediately speed-vac dried at 45°C and kept at -80°C. One μg of each sample was analyzed in an LTQ-Orbitrap Fusion Lumos mass spectrometer coupled to an EASY-nLC 1200 (Thermo Fisher Scientific) using a 90 min gradient at the Proteomic Unit of the Genomic Research Center (Barcelona, Spain). As a quality control, BSA controls were digested in parallel and ran between each sample to avoid carryover and assess the instrument performance.

### Protein Identification

Acquired spectra were analyzed using the Proteome Discoverer software suite (v2.0, Thermo Fisher Scientific) and the Mascot search engine (v2.6, Matrix Science). The data were searched against a customized database including *P. vivax* (all strains: 52920 entries) and Swiss-Prot human (20581 entries) (February 2020) plus a list of common contaminants and all the corresponding decoy entries. Peptides have been filtered based on a maximum false discovery rate (FDR) of 1% for peptides, one unique peptide per protein and a minimum peptide length of 8. Mass spectrometry proteomic data have been deposited to the ProteomeXchange Consortium *via* PRIDE (Vizcaino et al., 2016) with the dataset identifier PXD029439.

### Data Analysis

Protein groups identified in plasma-derived CD71^+^ EVs isolated by DIC were classified according to species *H. sapiens* and *P. vivax*. *H. sapiens* proteins were screened for common contaminants (keratins), immunoglobulins, complement proteins as well as potential serum contaminants (De Menezes-Neto et al., 2015) which were excluded from the analysis. *H. sapiens* protein groups identified with one unique peptide or more with a false discovery rate < 1% at peptide level and assigned with the category of Master protein by Proteome Discoverer™ software, were retained. The data have been transformed to a logarithmic scale (Log2) and have been normalized (Global median normalization). A statistical analysis at the protein group level was performed comparing the group of *P. vivax* patients versus non-infected patients. For each protein, we report the fold change, p-value and adjusted p-value (q-value). When a protein was detected in 5-6 patients in one group and it was not detected in the other group, we artificially assign it a *p*-value = 0 and a FoldChange = Infinite (they have been considered “presence versus absence”). Enrichment analysis of human proteins with statistical differential abundance in *P. vivax* infected patients was done with the Database for Annotation, Visualization, and Integrated Discovery (David 6.8) (Huang da et al., 2009). Results from the Gene ontology and KEGG databases are shown. *P. vivax* protein groups identified with one unique peptide or more, FDR < 1%, category of Master Protein by Proteome Discoverer software, were retained. Parasite protein groups identified in the group of healthy donors were excluded as false positives. Uniprot accession numbers of *P. vivax* protein groups were used to retrieve protein sequences and used to identify respective ID in Sal I and PVP01 genome through Blast analysis in PlasmoDB. Redundant entries were filtered out from the final list of proteins. Unresolved protein groups (> 2 accession numbers assigned) which were excluded by the algorithm (due to the redundancy of the customized database) were manually recovered. For *P. vivax* proteins subcellular localizations were retrieved from Uniprot. For proteins without subcellular localization data, predictions were performed based on orthology to *P. falciparum* or homologues in human proteins.

### Measurement of IgG Responses by Multiplex Bead-Based Assays (Luminex)

Measurement of plasma IgG antibodies was performed by multiplex bead-based assay using the Luminex technology, as described (Rui et al., 2011). The different recombinant proteins and peptides included in this study were covalently coupled to four MagPlex magnetic carboxylated microspheres (Luminex Corporation, TX, USA). Next, in 1.25 million coated beads we coupled 1 μg of each antigen following manufacturer’s instructions. Using a Neubauer chamber coated-beads were quantified and mixed in equal amounts. Only one batch of microspheres was prepared for the study. Plasma (1:100 dilution) was incubated with around 2000 beads per analyte in duplicates, followed by anti-human IgG-biotin (1:4000 dilution) (Sigma-Aldrich) incubation. Next, streptavidin-conjugated to R-phycoerthrin (R-PE) (1 μg/mL) was incubated for 10 min at RT and beads were acquired on the BioPlex100 system (Bio-Rad, Hercules, CA) and express the results as median fluorescence intensity (MFI) of duplicates. Cross-reactivity was ruled out analyzing a subset of plasmas in singleplex and multiplex. A panel of eight negative controls from healthy individuals from Barcelona was included on every plate. Cutoffs for antibody positivity were determined for each plate by calculating mean +2 standard deviation of the negative control values.

### Statistical Analysis

All data were analyzed using GraphPad Prism software (Version 8.3.0) and are presented as mean ± standard deviation. **Figure 2A** was a single analysis of *Pv*EVs (n=6) vs *hu*EVs (n=6) in which a Mann-Whitney non-parametric test was used to calculate the *p*-value represented in this experiment. Differences were considered significant when the p-value was < 0.05. *p < 0.05, **p < 0.01.

## Results

### Direct Immuno-Affinity Capture (DIC) Enrich CD71^+^ EVs From Plasma of *Plasmodium vivax* Patients

**Figure 1:**
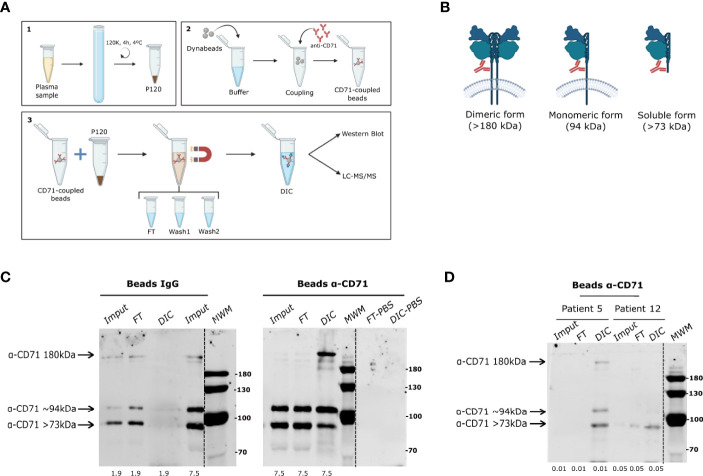
CD71 direct immuno-affinity capture (DIC). **(A)** Chart showing an overview of the direct immune-capture (DIC) procedure. 1. Generation of total EVs (P120 fraction) by ultracentrifugation. 2. Coupling of monoclonal anti-CD71 antibody to commercial dynabeads. 3. Direct immune-capture of CD71^+^ EVs from total plasma EVs indicating downstream analysis by western blot and LC-MS/MS. **(B)** Representation of oligomeric (membrane bound) and cleaved (soluble) CD71 forms with their respective molecular weights indicating the region of the epitope to which monoclonal anti-CD71 antibody binds. **(C)** Western blot analysis of exosomes immune-captured from culture supernatant of human reticulocytes isolated from cord blood using anti-CD71 dynabeads. Left panel shows absence of CD71 forms in the DIC fraction of the specificity control condition (rabbit IgG isotype-dynabeads). Right panel shows CD71 forms enriched in the DIC fraction of anti-CD71 dynabeads as compared to equivalent protein amount loaded from the input (µg). **(D)** Western blot analysis of CD71^+^ EVs immune-captured from total EVs (P120) of two *P. vivax* patients using anti-CD71-dynabeads showing enrichment of all forms of CD71 in patient 5 and only 73 kDa in patient 12. Dashed lines in the blots indicate a non-adjacent well from the same membrane incubated with anti-CD71 antibody. Protein amount loaded on the gels is shown at the bottom. MWM, molecular weight marker in kDa.

Reticulocytes, the host cell of *P. vivax*, actively and specifically secrete EVs containing CD71 (the transferrin receptor) in their maturation to reticulocytes (Harding et al., 1983; Pan and Johnstone, 1983). Here, we used anti-CD71 antibodies coupled to magnetic beads to immune-capture reticulocytes-derived EVs from total plasma EVs pre-enriched by ultracentrifugation (P120) (**Figure 1A**). Initially, we standardized the DIC technique using supernatants (SN) obtained from *in vitro* cultures of human reticulocytes (Diaz-Varela et al., 2018). In parallel, we coupled CD71 antibodies and rabbit IgG isotype to dynabeds as specificity control. In this context, it is important to recall that three forms of CD71 [Membrane bound dimeric (>180 kDa), monomeric (~94 kDa), and secreted soluble (>73 kDa) forms], are released from the plasma membrane (**Figure 1B**). Western blot analysis of immune-captured EVs from reticulocyte SN, showed that in DIC fractions using beads coupled to rabbit IgG, no CD71 forms were detected (**Figure 1C**). However, a clear enrichment in all forms of CD71 (notably the 180kDa form) was observed when SN from human reticulocytes culture were incubated with coupled anti-CD71-beads. No background detection of unspecific bands was observed when anti-CD71-beads were incubated with PBS.

Pre-Screening of CD71 in P120 fractions from ten *P. vivax* patients (**Supplementary Figure 1A**) by western blot (**Supplementary Figure 1B**), confirmed the detection of the three forms of CD71. A higher apparent molecular weight size was observed in all forms of the receptor likely associated to previously reported post-translational modifications (Do and Cummings, 1992). Western blot analysis of P120 fractions from HDs showed homogenous levels of CD71 among the donors with low abundance of the 94 kDa and 180 kDa forms (**Supplementary Figure 1C**). Yet, we observed a broad distribution in the abundance of the different forms of the receptor among the patients (**Supplementary Figure 1C**). This broad distribution was not correlated with clinical features such as parasitemia or previous malaria infections (data not shown). Based on the abundance of membrane-bound forms of CD71, we selected six *P. vivax* patients and six HDs to perform CD71^+^ EVs immune-capture. When immune-complexes were analyzed by western blot we found that anti-CD71 beads successfully captured and enriched CD71 forms from P120 fractions (**Figure 1D**). Together, these results demonstrate that CD71-DIC allows the specific enrichment of EVs containing all conformational forms of CD71 from reticulocytes culture SN and from the plasma of *P. vivax* malaria patients.

### The Human Proteome of CD71^+^ EVs From Plasma of *P. vivax* Patients Is Enriched in Proteins Involved in Malaria Pathogenesis

**Figure 2:**
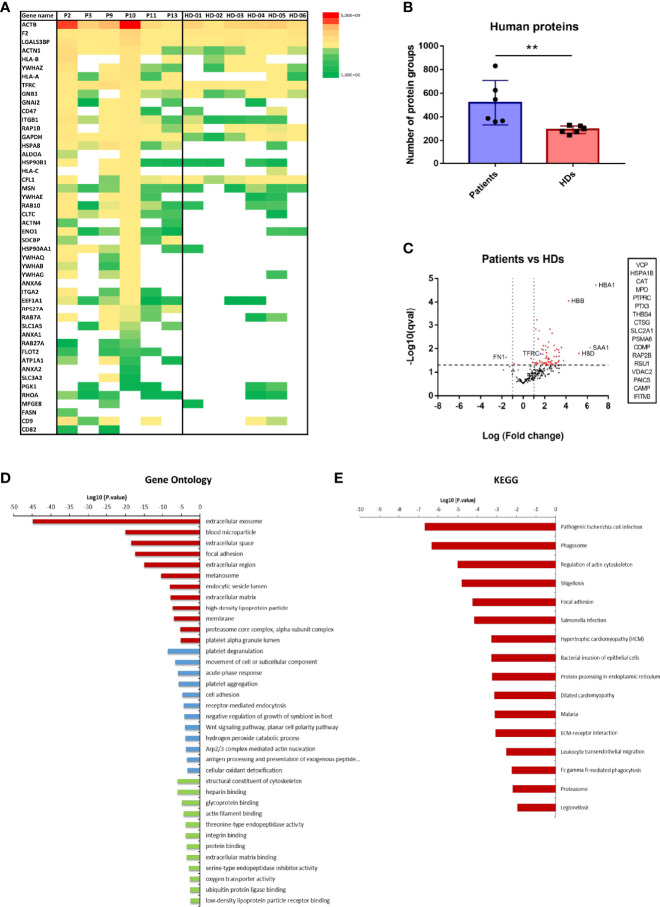
Human proteome of CD71^+^ EVs isolated by direct immuno-affinity capture (DIC) from *P. vivax* patients and healthy donors (HDs). **(A)** EV markers distribution and abundance in patients and healthy donor groups. Cytoskeleton proteins (ACTN1, ACTN4, ACTB, MSN and RHOA), members of the Annexin family (ANXA1, ANXA2, ANXA6), cell signaling regulators (YWHAB, YWHAE, YWHAG, YWHAQ and YWHAZ), integrins (ITGB1 and LGALS3BP), molecular chaperones (HSP90AA1 and HSPA8), glycolytic enzymes (GAPDH, ALDOA, and ENO1), GTP-binding proteins (RAP1B), GTPases (RAB10, RAB7A, RAB27A), Guanine nucleotide-binding proteins (GNB1 and GNAI2), regulators of lipid synthesis (CD5L), antigen presenting proteins (HLA-A), transmembrane trafficking proteins (SDCBP), iron uptake (TFRC) and members of the tetraspanin family (CD9) among others. **(B)** Mean of the total number of human protein groups quantified in *P. vivax* patients (n=6) and healthy donors (n=6). Mann-Whitney test,** p <0.05. **(C)** Quantitative comparison of protein groups abundance of CD71^+^ EVs from *P. vivax* patients and healthy donors. Volcano plot representation showing proteins up and downregulated (statistical significance q-val <0.05). Proteins listed in the inset were uniquely identified in *P. vivax* patients. **(D, E)** Enrichment analysis of proteins upregulated in CD71^+^ EVs from *P. vivax* patients by Gene ontology (GO) **(D)** and KEGG **(E)** pathway analysis. GO enrichment analysis shows terms of Cellular component (red), Biological process (blue) and Molecular function (green). **Supplementary Excel Files 1, 2** contain extended supporting data of these analysis.

Overall, 440 different human protein groups were identified in CD71^+^ EVs from patients and healthy donors (**Supplementary Excel File 1**). Initially, we confirmed the presence of CD71 in all MS-datasets. Remarkably, 30 unique CD71 peptides were identified among all samples and in those obtained from *P. vivax* patients we detected higher number and intensities of peptides (**Supplementary Figure 2A**). Moreover, 10% of detected peptides covered the cytoplasmic region (1 to 67 aa) and 90% the extracellular domain (89-760 aa) (**Supplementary Figures 2B, C**). Next, from a compiled list of 85 EV markers previously identified by mass spectrometry and isolated through different methodologies (De Menezes-Neto et al., 2015; Kowal et al., 2016; Jeppesen et al., 2019; Kugeratski et al., 2021), we identified 48 EV markers (**Figure 2A**). Of note, ACTB, F2, LGALS3BP, ACTN1 and the transferrin receptor protein (CD71/TFRC), were identified in all samples.

In order to explore possible CD71^+^ EVs human proteins associated to *P. vivax* infection, we performed a quantitative analysis of the abundance of all human protein groups in EVs from *P. vivax* patients compared to HDs. We observed a significant higher number of human protein groups associated with EVs from patients than those from HDs (**Figure 2B**). Remarkably, from 440 quantified protein groups, 73 showed statistically significant differential abundance in CD71^+^ EVs (q-value <0.05) with 97% of proteins upregulated in patients (**Figure 2C**). Only Fibronectin (FN1) and Coagulation factor XI (F11), two proteins involved in cell adhesion and motility, wound healing and coagulation were found downregulated (1.6 and 0.8-fold, respectively) in *P. vivax* patients derived CD71^+^ EVs (**Figure 2C**). Moreover, our analysis showed that 17 proteins were exclusively detected in CD71^+^ EVs from patients including proteins related with Golgi fragmentation, chaperones, oxidative stress, cell-to-cell and cell-to-matrix interactions, glucose transport, and inflammation among others (**Figure 2C**). The most highly abundant proteins in CD71^+^ EVs from patients included proteins involved in oxygen transport [Hemoglobin subunit alpha, beta and delta (HBA1, HBB, HBD)] and acute phase response to infection [Serum amyloid A-1 protein (SAA1), major acute phase protein] with 4 to 6-fold increase compared to HD EVs.

Functional enrichment analysis of proteins upregulated in CD71^+^ EVs from patients by gene ontology showed enriched cellular component related to extracellular vesicles, blood microparticle and extracellular space and enriched biological processes related to platelet degranulation, movement of cell or subcellular component, cell adhesion and antigen presentation, among others (**Figure 2D** and **Supplementary Excel File 2**). In addition, KEGG pathway analysis indicate the enrichment of proteins related to bacterial and malarial infections, protein processing in endoplasmic reticulum and phagosome, among others (**Figure 2E** and **Supplementary Excel File 2**).

### Circulating CD71^+^ EVs Obtained From Natural Infections Contain Diverse Parasite Proteins

**Figure 3:**
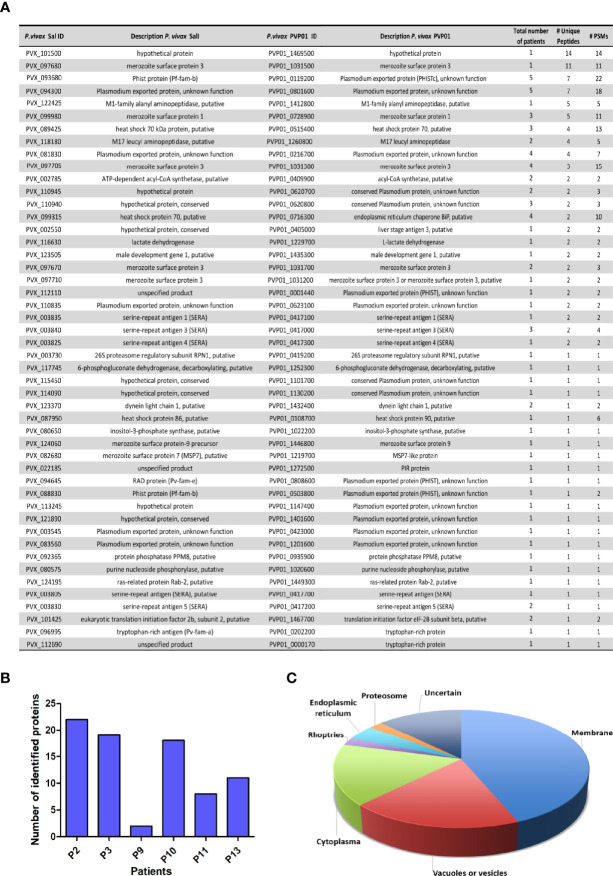
*P. vivax* protein groups identified in proteomic analysis of CD71^+^ EVs isolated by direct immuno-affinity capture (DIC) from plasma of *P. vivax* patients. **(A)** Non-redundant *P. vivax* protein groups quantified in CD71^+^ EVs indicating Sal-I and PVP01 PlasmoDB accessions and descriptions, number of samples where they were identified, number of unique peptides and PSMs. Extended supporting data of these analysis can be found in **Supplementary Excel File 1**. **(B)** Parasite protein groups distribution in *P. vivax* patients. **(C)** Pie chart showing predicted subcellular localization of parasite protein groups identified in CD71^+^ EVs. Subcellular compartment assignations were retrieved from Uniprot. In those cases where localization was unknown, predictions were performed by inference in orthologues of *P. falciparum* or homologues in human.

Remarkably, unlike our previous results where we detected with confidence three parasite proteins associated with circulating EVs isolated by SEC in two out of ten patients (Toda et al., 2020), the proteomic analysis of CD71^+^ EVs using CD71-DIC identified parasite proteins in all patients, totaling 48 parasite protein groups (**Figure 3A**). The distribution of these proteins in the different patients, however, indicate high variability among them with three out of six containing an average of 20 parasite proteins while others less than 10 parasite proteins (**Figure 3B**). Importantly, we did not find a correlation between the number of parasite proteins quantified and the abundance of CD71 (any of the forms) in the starting P120 fraction (data not shown). Subcellular localization analysis of parasite proteins in CD71^+^ EVs indicate that 42% were predicted to be associated to diverse parasite membranes, 19% in vacuoles or vesicles, 15% in the cytoplasm, 4% in endoplasmic reticulum (ER), 2% in proteasome, 2% in rhoptries and for the remaining proteins their localization was undetermined (**Figure 3C**). Noticeably, 24 proteins were identified with >2 unique peptides, thus robustly indicating their presence in EVs enriched by CD71-DIC (**Supplementary Excel File 1**). The most abundant parasite proteins correspond to a hypothetical protein, the merozoite surface protein 3.3 and a member of the PHIST family, PHISTc. Other parasite proteins associated to CD71^+^ EVs are merozoite surface proteins, heat shock proteins, serine-repeat antigen 1 and PHIST proteins. In addition, we also identified 11 proteins of unknown function from which 7 corresponded to predicted plasmodium exported proteins. Altogether, the proteomic analysis of circulating CD71^+^ EVs from *P. vivax* patients robustly identified a set of parasite proteins from different membranous compartments in agreement with its physical interaction with EVs.

### Humoral Immune Response Against *P. vivax* Proteins Discovered in CD71^+^-EVs

Members of the MSP3 family and PHISTc were among the most abundant parasite proteins identified in the proteome of CD71^+^ EVs. Antigenicity of both of these proteins have been previously reported (Bitencourt et al., 2013; Lu et al., 2014). In order to expand these results, we analyzed antibody response (IgG levels) of primo-infected *P. vivax* patients coming to Barcelona (travelers returning from endemic areas) against purified recombinant versions of MSP3.1 and PHISTc by Luminex technique. Antibody responses were detected above negative control cut-off for MSP-3 and PHIST recombinant *P. vivax* proteins in three out of four patients while for MSP-19 recombinant protein antibody responses were detected in all patients (**Supplementary Figure 3**).

## Discussion

The development of a vaccine against *Plasmodium vivax*, the most widely distributed human malaria parasite, remains a formidable challenge as only two vaccine candidates are presently in clinical trials (Draper et al., 2018). Moreover, other vaccine candidates progressing to human clinical trials are based on a limited number of parasite antigens. Although new approaches and delivery platform are being pursued (De et al., 2021), there is a clear need for discovering new antigens for vaccination.

The biological properties of EVs endorse them with a remarkable potential as new antigen discovery machines and novel vaccines against infectious diseases. Thus, original data obtained from exosomes derived from dendritic cells (DCs) infected with *Mycobacterium avium*, an opportunistic parasite in AIDS patients, contained pathogen glycopeptidolipids and stimulated a pro-inflammatory response in resting non-infected macrophages (Bhatnagar and Schorey, 2007). Similar observations were also made with exosomes derived from macrophages infected with other intracellular pathogens, *Mycobacterium tuberculosis*, *M. bovis* BCG, *Salmonella typhimurium* and *Toxoplasma gondii* (Bhatnagar et al., 2007). More recently, proteomic studies have confirmed the presence of pathogen proteins associated with EVs in different infectious diseases (Lee et al., 2009; Lee et al., 2015; Wowk et al., 2017). Of interest, plasma-derived exosomes obtained from pigs that had overcome an infection caused by the Porcine Reproductive and Respiratory Syndrome Virus (PRRSV), a single-strand RNA virus, that induces respiratory failure similarly to Sars-Cov-2, demonstrated the presence of viral peptides associated to EVs which upon immunization in targeted-pig trials, elicited specific γ-IFN responses (Montaner-Tarbes et al., 2018). In the case of malaria, original studies using a rodent malaria model that, similar to *P. vivax* has a tropism for reticulocytes, showed that reticulocyte-derived exosomes from infections conferred long lasting protection against a lethal challenge (Martin-Jaular et al., 2011). Protection was spleen-dependent and elicited non-exhausted memory T cells with effector phenotype (Martin-Jaular et al., 2016). Altogether, the data above clearly show that extracellular vesicles obtained from different infectious diseases, including malaria, can discover antigens for vaccination and can be used as an antigen delivery platform.

Our overall proteomic analysis of CD71^+^ EVs obtained directly from human *P. vivax* patients showed the presence of 440 human proteins groups including 48 EVs markers and 48 parasite proteins (**Supplementary Table 1**). Among the EV markers, CD71 (TFRC), was detected in all of the samples with 30 unique peptides (27 mapping to extracellular domains of CD71 and 3 mapping to the cytoplasmic tail). These data strongly indicate that immune-captured CD71 corresponds to membrane bound monomeric and/or dimeric forms. Due to the abundance of soluble CD71 in plasma and that preparative ultracentrifugation does not eliminate completely abundant plasma protein from the starting material, we cannot exclude that non-EV associated CD71 soluble form will also be pull down in the immune-captures. Yet, other EV markers include canonical EVs associated proteins such CD9, Flotillin-2, HSPA8, HSP90AA1B as well as 13 out of 22 core proteome exosomal markers recently described (Kugeratski et al., 2021). Together, these results strongly suggest that immune-captured CD71^+^ EVs from human plasma are mostly comprised of exosomal vesicles.

The methodology employed here allowed the detection of 48 *P. vivax* proteins in CD71^+^ EVs, 28 of them robustly detected with two or more unique peptides (**Figure 3A**). These proteome data contrast with our previous report of EVs isolated by SEC, indicating a superior performance of this method for the identification and quantification of parasite proteins in plasma-derived EVs. The vast majority of detected *P. vivax* proteins (**Figure 3C**) correspond to proteins associated to membranes, some well stabilized surface anchored proteins like several members of the merozoite surface family (MSP1, MSP3, MSP7, MSP9), an integral membrane VIR protein (PVP01_1272500), four members of the PHIST family, and five members of the Plasmodium exported protein family, both families observed in five patients and one of which (PVX_094300) detected in five patients, and some (PVX_081830, PVX_110835 and PVX_083560) having orthologues of conserved *P. falciparum* proteins of unknown function. Proteins from other vesicular compartments like lysosomes included several members of the serine repeat antigens, as well as proteins located in the Golgi apparatus (Ras-related protein Rab-2 putative) and a PvFam-a protein (Tryptophan rich antigen) which orthologue in *P. falciparum* has been located in Maurer’s cleft (MC). Overall, the presence of abundant membrane associated parasite proteins is in agreement with its potential interaction with host-derived exosomes.

**Figure 4:**
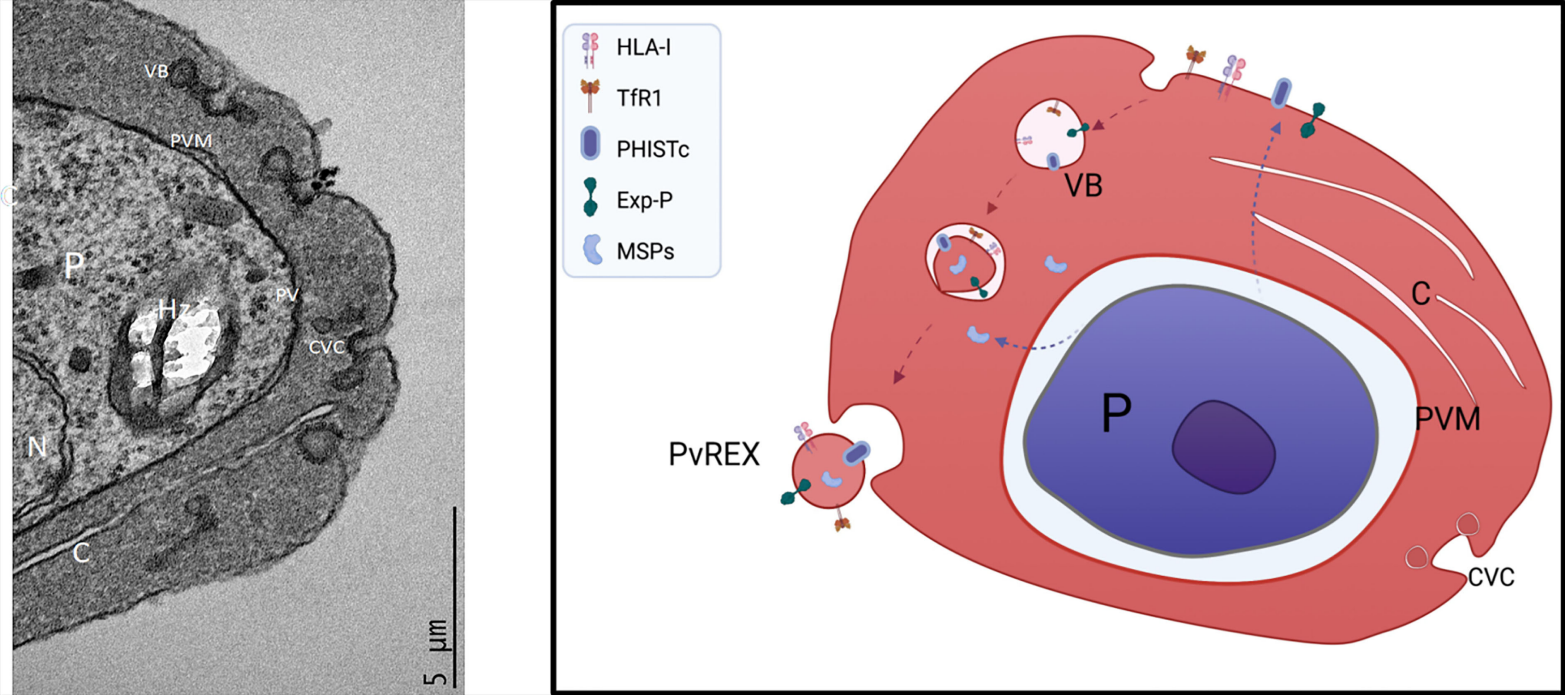
Model of EV biogenesis in *P. vivax*-Infected reticulocytes. Left, transmission electron micrograph (TEM) image of a *P. vivax*-infected reticulocyte. P, parasite; N, nucleus; Hz, hemozooin crystal; PV, parasitophorous vacuole; PVM, parasitophorous vacuole membrane; VB, Vesicular body; C, Clefts; CVC, Caveola-Vesicle-Complex. Right. Model of EV biogenesis. Upon entrance to the reticulocyte, *P. vivax* remodels the cytoplasm, including the formation of clefts facilitating the export of variant proteins such as PHISTc and *Plasmodium* exported proteins to the reticulocyte membrane. Endocytosis of this membrane containing parasite proteins, the transferrin receptor (CD71), and other selected cargo (i.e. HLA-I), creates a single membrane cytoplasmic endosome. Upon re-invagination of this membrane and incorporation of soluble proteins found in the cytoplasm, electrodense double membrane vesicles are formed which upon fusion to CVC release CD71^+^ EVs. TEM image and model was made in biorender contributed by Carmen Fernandez-Becerra.

The biogenesis of EVs in *P. vivax* is presently unknown. Yet, this species has a tropism for reticulocytes, young red cells which selectively removes plasma proteins [noticeably the transferrin receptor (CD71)] in their maturation to erythrocytes (Harding et al., 1983; Pan and Johnstone, 1983). Elegant transmission electron microscope (TEM) studies showed that upon entrance of the parasite, and similar to *P. falciparum*, there is a striking remodeling of the cytoplasm containing the parasite and its membrane inside a parasitophorous vacuole (PV) also surrounded by a PV membrane (PVM), clefts and electrodense vesicular bodies (Aikawa et al., 1975). Strikingly, unlike *P. falciparum* which forms protrusions at the plasma membrane where variant surface virulent determinants are located, *P. vivax* induced the formation of Caveola-Vesicle-Complex (CVC) where parasite proteins are located (Aikawa et al., 1975; Akinyi et al., 2012). Further TEM evidence of this remodeling and structures has been obtained by us as part of another study (**Figure 4**). It is therefore legitimate to speculate that upon entrance to the reticulocyte, *P. vivax* remodels the cytoplasm, including the formation of clefts facilitating the export of variant proteins such as PHISTc and *Plasmodium* exported proteins to the reticulocyte plasma membrane. Endocytosis of this membrane containing parasite proteins, the transferrin receptor (CD71), and other selected cargo (i.e. HLA-I), creates a single membrane cytoplasmic endosome. Upon re-invagination of this membrane and incorporation of soluble proteins found in the cytoplasm, electrodense double membrane vesicles are formed which upon fusion to CVC release CD71^+^ EVs.

Noticeably, several of the identified parasite proteins are immunogenic; thus, the merozoite surface protein 1 (PVX_099980) (Fernandez-Becerra et al., 2010), the heat shock 70 kDa protein, putative (PVX_089425) (Na et al., 2007), the serine-repeat antigen 1 (SERA) (PVX_003835) (Son et al., 2001), the merozoite surface protein-9 precursor (PVX_124060) (Oliveira-Ferreira et al., 2004), the merozoite surface protein 7 (MSP7), the putative (PVX_082680) (Lee et al., 2019) and the PHIST protein (Pf-fam-b) (PVX_088830) (Lu et al., 2014). These data strongly indicate that parasite proteins associated with CD71^+^ EVs are antigenic. In this context, we found in CD71^+^ EVs sixteen protein groups associated to the antigen processing and presentation of exogenous peptide antigen via MHC class I, TAP-dependent including all three isoforms of the major histocompatibility complex class I (HLA-A, HLA-B, HLA-C), β-2 microglobulin, CD36, integrin subunit beta 5 (ITGB5) and 9, proteosome subunit alpha (1-7) and proteosome subunit B (1-2). These data indicate that plasma-derived exosomes from *P. vivax* patients contain novel antigens for vaccination and that they can be further explored as a novel antigen delivery platform.

## Data Availability Statement

The datasets presented in this study can be found in online repositories. The names of the repository/repositories and accession number(s) can be found in the article/**Supplementary Material**.

## Ethics Statement

Plasma samples from *P. vivax*-infected patients (PV) were collected at Tierralta, Colombia. The local ethical committee of Universidad de Córdoba reviewed and approved these studies (Acta #007 mayo 22 de 2017). Plasma from healthy donors (HD) was obtained at the Hospital Germans Trias i Pujol (Badalona, Barcelona, Spain) after expressed consent from the donors. Plasma from first-infected *P. vivax* patients from Barcelona was collected at the Hospital Clinic under the approval of the Hospital´s committee (identification number 4228). The patients/participants provided their written informed consent to participate in this study.

## Author Contributions

IAH, MG-L, and CC-C performed experiments. MG-L performed the proteomics analysis. MY and JC-F contributed materials. IA, MG-L, and HP drafted the manuscript. CF-B contributed TEM image. IAH, MG-L, CF-B, and HP originally conceived and designed the study. All authors contributed to the article and approved the submitted version.

## Funding

MG-L is a postdoctoral fellow supported by ISGlobal. IAH is a predoctoral fellow supported by the Ministerio de Economia y Competitividad (FPI BES-2017081657). We acknowledge support from the Spanish Ministry of Science and Innovation through the Centro de Excelencia Severo Ochoa 2019-2023 Program (CEX2018- 000806-S), and support from the Generalitat de Catalunya through the CERCA Program. This research is part of the ISGlobal’s Program on the Molecular Mechanisms of Malaria which is partially supported by the Fundación Ramón Areces. ISGlobal and IGTP are members of the CERCA Programme, Generalitat de Catalunya. CIBERINFEC is co-funded with FEDER funds. Work in the laboratory of CF-B and HP is funded by the Ministerio Español de Economía y Competitividad (SAF2016 80655-R) and by the Ministerio de Ciencia e Innovación (PID2019-111795RB-I00).

## Conflict of Interest

The authors declare that the research was conducted in the absence of any commercial or financial relationships that could be construed as a potential conflict of interest.

## Publisher’s Note

All claims expressed in this article are solely those of the authors and do not necessarily represent those of their affiliated organizations, or those of the publisher, the editors and the reviewers. Any product that may be evaluated in this article, or claim that may be made by its manufacturer, is not guaranteed or endorsed by the publisher.

## Supplementary Material

Supplementary Figure 1 CD71 abundance in total plasma-derived EVs in vivax malaria patients. **(A)** Clinical data of patients enrolled in the study. Data include age, gender, parasitemia, previous malaria infections, pregnancy and time or residence in the marginal zone (months). **(B, C)** Pre-screening of CD71 in total EVs (P120 fraction) from patients (n=12, **B**) and healthy donors (n=6, **C**). Fifty µg of protein were loaded in each well. Human reticulocyte-derived exosomes (huRex) were used as positive control of CD71^+^ EVs. * Selected patients for proteomics analysis (n=6).

Supplementary Figure 2 Transferrin receptor (TFRC) detection in CD71^+^ EVs by LC-MS/MS. **(A)** Mass spectrometry data of TFRC detection showing sequence, intensity and positions of the 30 CD71 unique peptides detected in CD71^+^ EVs from patients and healthy donors. **(B)** TFRC topology indicating the amino-acids position of intracellular, transmembrane and extracellular regions. **(C)** Pie chart showing peptides coverage of CD71 cytoplasmic and extracellular regions in the CD71^+^ EVs mass spectrometry analysis.

Supplementary Figure 3 Humoral immune response of vivax malaria primo-infected individuals against three abundant antigens detected in CD71^+^ EVs. Luminex analysis showing antibody levels [represented as mean fluorescence intensity (MFI)] against three antigens (MSP-19, MSP-3, and PHIST). Dotted lines represent cut-off point established for each antigen by calculating the mean +2 standard deviation of the signal obtained in healthy controls (n=8) used as negative controls. Pv, *P. vivax*; BCN, Barcelona.
