# Supplementary figures and images for: Antigen Discovery in Circulating Extracellular Vesicles From Plasmodium vivax Patients
Source: Front Cell Infect Microbiol. 2022 Jan 24;11:811390. doi: 10.3389/fcimb.2021.811390 (PMC8819181; doi:10.3389/fcimb.2021.811390)

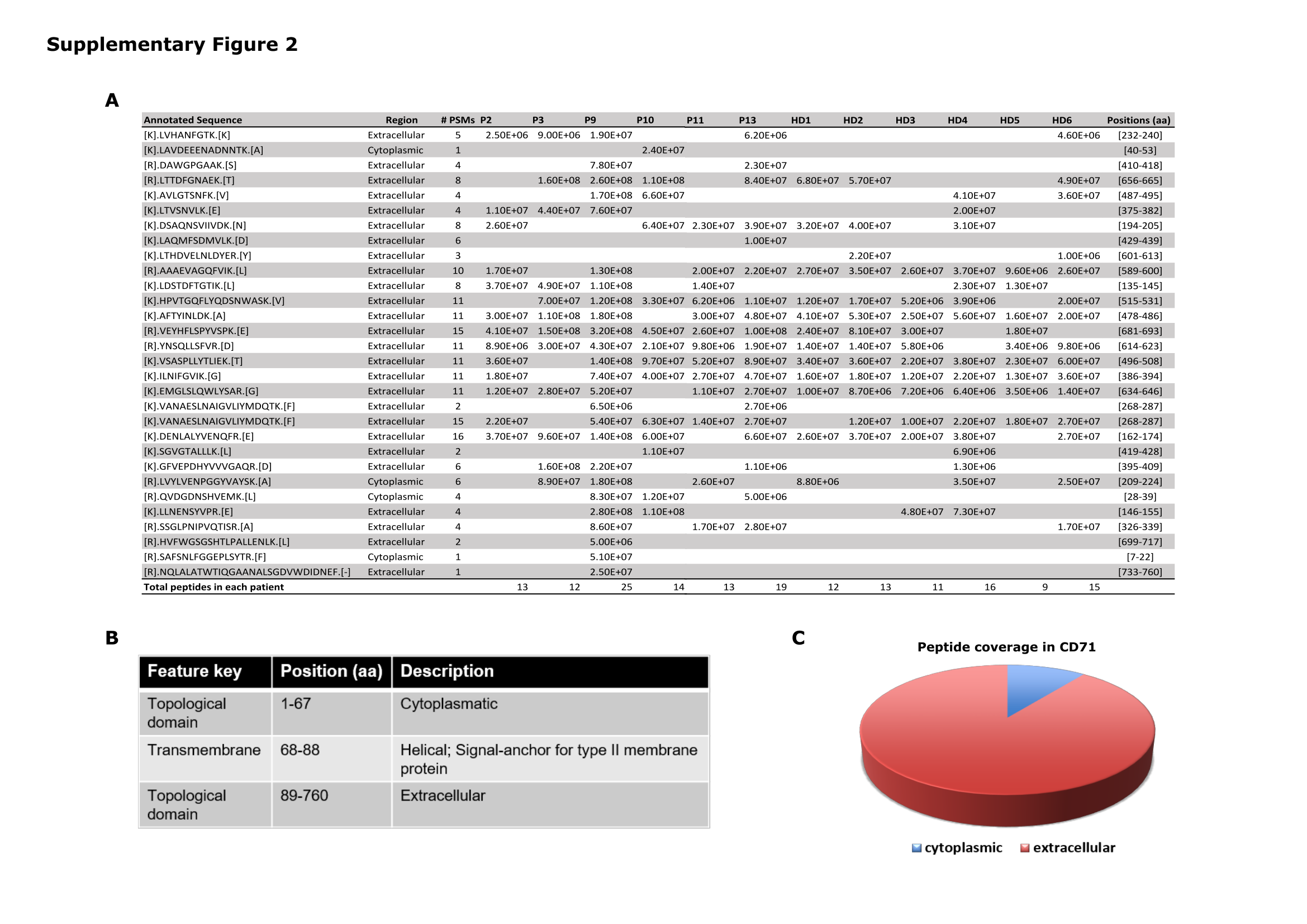

Supplement: Supplementary Figure 2 — Transferrin receptor (TFRC) detection in CD71+ EVs by LC-MS/MS. (A) Mass spectrometry data of TFRC detection showing sequence, intensity and positions of the 30 CD71 unique peptides detected in CD71+ EVs from patients and healthy donors. (B) TFRC topology indicating the amino-acids position of intracellular, transmembrane and extracellular regions. (C) Pie chart showing peptides coverage of CD71 cytoplasmic and extracellular regions in the CD71+ EVs mass spectrometry analysis. [file Image_2.tiff]

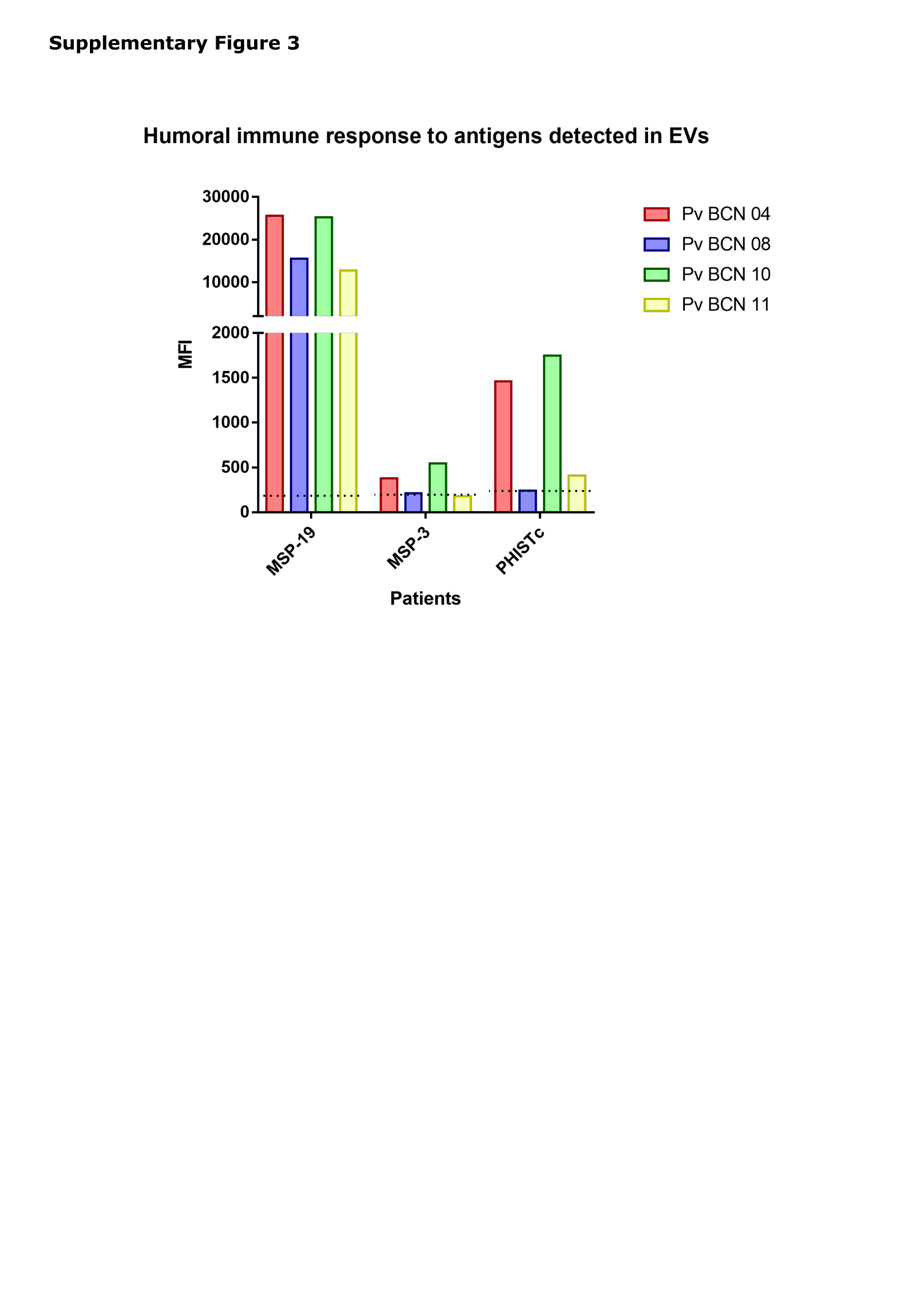

Supplement: Supplementary Figure 3 — Humoral immune response of vivax malaria primo-infected individuals against three abundant antigens detected in CD71+ EVs. Luminex analysis showing antibody levels [represented as mean fluorescence intensity (MFI)] against three antigens (MSP-19, MSP-3, and PHIST). Dotted lines represent cut-off point established for each antigen by calculating the mean +2 standard deviation of the signal obtained in healthy controls (n=8) used as negative controls. Pv, P. vivax; BCN, Barcelona. [file Image_3.tiff]
